# Protein-Mediated Carotenoid Delivery Suppresses the Photoinducible Oxidation of Lipofuscin in Retinal Pigment Epithelial Cells

**DOI:** 10.3390/antiox12020413

**Published:** 2023-02-08

**Authors:** Alexey N. Semenov, Eugene G. Maksimov, Anastasia M. Moysenovich, Marina A. Yakovleva, Georgy V. Tsoraev, Alla A. Ramonova, Evgeny A. Shirshin, Nikolai N. Sluchanko, Tatiana B. Feldman, Andrew B. Rubin, Mikhail P. Kirpichnikov, Mikhail A. Ostrovsky

**Affiliations:** 1 Faculty of Biology, M.V. Lomonosov Moscow State University, 1-12 Leninskie Gory, 119991 Moscow, Russia; 2 Interdisciplinary Scientific and Educational School “Molecular Technologies of the Living Systems and Synthetic Biology”, M.V. Lomonosov Moscow State University, 1 Leninskie Gory, 119991 Moscow, Russia; 3 Emanuel Institute of Biochemical Physics, Russian Academy of Sciences, 4 Kosygin Str., 119334 Moscow, Russia; 4 Faculty of Physics, M.V. Lomonosov Moscow State University, 1-2 Leninskie Gory, 119991 Moscow, Russia; 5 World-Class Research Center “Digital Biodesign and Personalized Healthcare”, Sechenov First Moscow State Medical University, 8-2 Trubetskaya Str., 119991 Moscow, Russia; 6 Institute of Spectroscopy of the Russian Academy of Sciences, 5 Fizicheskaya Str., 108840 Moscow, Russia; 7 Federal Research Center of Biotechnology, Russian Academy of Sciences, 33 Leninsky Prospect, 119071 Moscow, Russia

**Keywords:** carotenoid-binding protein, zeaxanthin delivery, retinal pigment epithelium, lipofuscin, bisretinoid fluorophores, bisretinoid oxidation and degradation products, FLIM

## Abstract

Lipofuscin of retinal pigment epithelium (RPE) cells is a complex heterogeneous system of chromophores which accumulates as granules during the cell’s lifespan. Lipofuscin serves as a source of various cytotoxic effects linked with oxidative stress. Several age-related eye diseases such as macular degeneration of the retina, as well as some severe inherited eye pathologies, are accompanied by a significant increase in lipofuscin granule concentration. The accumulation of carotenoids in the RPE could provide an effective antioxidant protection against lipofuscin cytotoxic manifestations. Given the highly lipophilic nature of carotenoids, their targeted delivery to the vulnerable tissues can potentially be assisted by special proteins. In this study, we demonstrate how protein-mediated delivery of zeaxanthin using water-soluble *Bombyx mori* carotenoid-binding protein (BmCBP-ZEA) suppresses the photoinducible oxidative stress in RPE cells caused by irradiation of lipofuscin with intense white light. We implemented fluorescence lifetime imaging of the RPE cell culture ARPE-19 fed with lipofuscin granules and then irradiated by white light with and without the addition of BmCBP-ZEA. We demonstrate that after irradiation the mean fluorescence lifetime of lipofuscin significantly increases, while the presence of BmCBP-ZEA at 200 nM concentration suppresses the increase in the average lifetime of lipofuscin fluorescence, indicating an approx. 35% inhibition of the oxidative stress. This phenomenon serves as indirect yet important evidence of the efficiency of the protein-mediated carotenoid delivery into pigment epithelium cells.

## 1. Introduction

The accumulation of lipofuscin granules inside retinal pigment epithelial (RPE) cells is generally described as an age-related pathology. The occurrence of lipofuscin granules is suggested to be the result of an incomplete lysosomal degradation of the fragments of the outer segments of photoreceptors during RPE cells’ lifespan [1]. For some time, lipofuscin was considered as an inert side-product of retinal cells’ metabolic activity, until its ability to form reactive oxygen species (ROS) when exposed to light [2] and the cytotoxic manifestations of its massive accumulation were clearly demonstrated [3,4,5]. Namely, such common age-related eye disease as macular degeneration of the retina is accompanied by a significant increase in lipofuscin granules intracellular concentration, which complicates the treatment dramatically, and without proper control may lead to an irreversible loss of vision [6,7]. Likewise, the abnormally increased accumulation of lipofuscin in RPE cells takes place in some inherited human ophthalmic pathologies such as Stargardt disease [8] and vitelliform macular degeneration (Best macular dystrophy) [9].

The structure and composition of lipofuscin granules inside RPE cells is not yet understood completely. In contrast to the lipofuscin of other tissue cells, RPE lipofuscin consists mainly of retinal derivatives with some presence of proteins specific to the outer segments of the rods, for example, recoverin [10]. Proteomic analysis of RPE lipofuscin revealed some additional non-specific protein components such as residues of cytoskeletal protein complexes, enzymes, chaperone proteins, and channel proteins [11]. The presence of such protein fragments indicates that the lipofuscin granules originate not only from the remains of photoreceptor machinery but also from the products of autophagocytosis of other RPE cell organelles.

The set of chromophores in RPE lipofuscin includes more than 20 components, with bisretinoids and their photo-oxidation and photodegradation products being the most abundant fraction [12]. Among them, N-retinylidene-N-retinylethanolamine (A2E), a pyridinium bisretinoid [13], has been identified and characterized in detail [14]. A2E is supposed to form in the reaction between two molecules of all-*trans*-retinal and membrane phospholipid phosphatidylethanolamine. The exact localization of this reaction is debatable [15]: present mechanisms suggest that A2E forms either inside the cell in the phagolysosome during autophagocytosis or outside the cell in the photoreceptor membrane with subsequent allocation in the cytoplasm as a part of the fragment of phagocytized outer segment debris. Studies have shown that singlet oxygen production, driven by A2E photosensibilization after irradiation in the visible region, leads to the formation of autoxidation products [2] which in a culture of RPE cells represents a source of massive free-radical cell damage [16]. Hence, the general mechanism of the lipofuscin photoinduced cytotoxicity suggests the involvement of the generation of ROS [2,5,17]. Thus, after irradiation, lipofuscin granules provoke massive oxidative stress resulting in the oxidation of lipids, non-specific cleavage of proteins or inactivation of their enzymatic activity, and biological membrane damage, ultimately resulting in apoptosis [18]. Oxidative stress caused by photic activation of lipofuscin significantly impairs microcirculation within the eye tissue, resulting in vascular dysfunction, which is suggested to be critically involved in macular degeneration [19]. Additionally, lipofuscin granules are commonly suggested to be responsible for the intensification of most of the degenerative processes of RPE cells, especially those that are caused by short-wave (violet-blue) visible light irradiation (“blue-light hazard”) [20,21].

The usage of antioxidants can prevent the harmful effects of the photoinduced toxicity of lipofuscin in RPE cells by inhibiting the autoxidation of bisretinoids and A2E in particular [22]. Such an effect was reported for anthocyanins [23,24] and vitamin E [25]. Similarly, it was reported that resveratrol, a naturally occurring polyphenol, decreased A2E-provoked oxidative damage in the RPE cell line ARPE-19 [26]. Due to their spectacular antioxidant and anti-inflammatory properties, xanthophylls, namely lutein (LUT) and zeaxanthin (ZEA), are proposed as potential therapeutic agents providing various beneficial effects in delaying the development of eye diseases such as age-related macular degeneration and cataracts [27]. The mechanisms of the protective effects of ZEA against photo-oxidation in RPE cells have been intensively studied. ZEA possesses a combination of various oxidant-protective effects. One mechanism suggests the direct suppression of A2E photo-oxidation by quenching singlet oxygen and lipid peroxidation radicals [28], with ZEA being more effective than LUT, possibly due to zeaxanthin’s more extensive double-bond conjugation [29]. A second possible direct mechanism suggests the ability of ZEA and LUT to filter high-energy short-wavelength blue light and thus decrease the A2E photo-oxidation level [30,31]. In [32], it was demonstrated that the enrichment of lipofuscin-fed RPE cells with ZEA impairs the specific phagocytic activity of the RPE and thus decreases further lipofuscin intracellular accumulation. An indirect protection effect of ZEA and LUT by suppressing the level of A2E production in RPE cells was reported [33], although the underlying mechanisms are not fully understood. A molecular interaction between carotenoids and photoreceptor cells can take place: recently it was reported that isomers of LUT and ZEA may modulate the expression of G-protein-coupled receptors (i.e., rhodopsin) in the photoreceptor membrane and also mediate growth factors in rats [34], decreasing light-induced inflammation and suppressing retinal degeneration.

Recently, attempts have been made to evaluate the protection effects of antioxidants against the lipofuscin photoreactivity in the RPE [35]. Due to their highly lipophilic nature, effective target delivery of ZEA and LUT in the aqueous environment of the human body is difficult. One of the possible approaches to overcome this obstacle is the application of transportation devices. In [36], the authors proposed to deliver LUT or ZEA to cultured human RPE cells using micelles prepared from various bio-organic solutions, i.e., tween-40. However, the choice of bio-organic solvents capable of dissolving carotenoids in this method is strictly limited since the retinal pigment epithelium is a very sensitive kind of tissue, especially in the case of pathology. The delivery of carotenoids using nanoemulsion liposomes is being actively discussed nowadays and is considered to have several advantages [37,38,39]. However, in the liposomal form, the incorporation preferences and release of carotenoids, as well as their storage stability and lipid peroxidation capacity, are limited and greatly depend on the carotenoid type [40,41]. A recently proposed approach involves using monoclonal antibodies for the target delivery of carotenoids [42]. It is a promising mechanism; however, the antibody preparation is very complicated and therefore expensive and still requires the use of organic solvents, in particular DMSO, to prepare the solution of the carotenoid. Thus, the research, development, and further usage of the delivery systems capable of carotenoid transport in aqueous media is sought after.

The carotenoid delivery strategy utilizing protein-mediated mechanisms creates new potentialities since it facilitates the intracellular transportation of carotenoids in the aqueous environment. In humans, proteins which play a role in the uptake, transport, and metabolism of fat-soluble compounds are capable of facilitating targeted carotenoid delivery. Scavenger receptor class B member 1 (SCARB1) and CD36 complex, integral membrane proteins found in numerous cell types and responsible for intracellular transport of cholesteryl esters and fatty acids, respectively, were reported to implement the membrane transport of carotenoids and several fat-soluble vitamins [43]. In [44], it was shown that carotenoids can be transported from the liver to the eye tissue by high-density lipoproteins (HDLs), and apolipoprotein A-I (ApoA-I) may be involved in the selective delivery of macular carotenoids to RPE cells. In the human retina, several proteins were identified as carotenoid-binding proteins, including a pi isoform of glutathione S-transferase (GSTP1) as a zeaxanthin-binding protein [45]; a member of the steroidogenic acute regulatory domain (StARD) family as a lutein-binding protein [46]; and tubulin as a less specific but high-capacity site for the carotenoid deposition [47]. The activity pattern of aster protein GRAMD1B, responsible for cholesterol transport, is also consistent with its role in carotenoid uptake [48]. In this way, protein-mediated mechanisms of carotenoid transport provide an opportunity to establish an efficient and noncytotoxic delivery of carotenoids into cells. Their use as a potential delivery platform for carotenoids has many advantages including wide application in various industries such as food biotechnology and pharmaceuticals.

Cyanobacterial water-soluble carotenoid-binding proteins, such as orange carotenoid protein (OCP) [49], are evolutionarily aimed at the implementation of the optimal uptake and transportation of carotenoids, as the latter play a key role in photoprotection in cyanobacteria [50,51]. However, the common use of OCP-related proteins is limited since they are capable of binding only specific carotenoids [52]. For example, the homologs of the N- and C-terminal domains of OCP are strictly capable of binding keto-carotenoids, and only the delivery of echinenone (ECN) to the cell membrane was proved to be effective in the inhibition of oxidative stress [53]. Nevertheless, new water-soluble carotenoproteins have recently been discovered, and their ability to bind and transport various types of carotenoids was characterized. In recent studies [52,54], a functional ~27 kDa carotenoid-binding protein, responsible for the coloration of silkworm *Bombyx mori* cocoons (BmCBP), was reconstituted, and its capability of concentration-dependent binding of LUT and ZEA was demonstrated. The potential of BmCBP for delivering carotenoids to model membranes was clearly demonstrated, as well as its ability to deliver ZEA to fibroblasts promoting their growth [54].

The aim of the present study was to investigate the efficiency of the protein-mediated delivery of ZEA into RPE cells by assessing the effects of BmCBP carotenoprotein in holoform on the photo-oxidation of lipofuscin granules. We utilized fluorescence microscopy, including fluorescence lifetime imaging (FLIM), to assess the changes in the state of lipofuscin granules inside RPE cells photo-oxidized after sufficiently intense white light irradiation. This allowed us to show that protein-mediated delivery of antioxidants is promising for protecting cells from lipofuscin-induced photodamage.

## 2. Materials and Methods

### 2.1. Isolation of the Lipofuscin Granules

Lipofuscin granules (LG) were isolated from the RPE cells from 85 human cadaver eyes without ophthalmologic pathologies according to the protocol described in [2]. Cadaver eyes were obtained from the Eye Tissue Bank of the S.N. Fyodorov Eye Microsurgery Complex (Moscow, Russia) within 10 h after donor death. Donor age ranged from 30 to 75 years. Collection of eyes was conducted in accordance with local ethics requirements as described in detail previously [55]. All stages of lipofuscin granule isolation were performed under subdued illumination to avoid undesirable photo-oxidation. The lipofuscin granule stock solution concentration, calculated in a Goryaev chamber, was 4 × 10^9^ granules/mL.

### 2.2. ARPE-19 Cell Preparation

#### 2.2.1. ARPE-19 Cell Line Culturing

Spontaneously arising retinal pigment epithelial cell line culture ARPE-19 was obtained from the Cell Culture Collection of the Koltzov Institute of Developmental Biology of Russian Academy of Sciences (Moscow, Russia). ARPE-19 cell suspension at a concentration of 0.5 × 10^6^ (cells/dishes) was cultured in supplemented culture media DMEM/F12 (Paneco, Moscow, Russia) containing 10% fetal bovine serum (HyClone GE Healthcare, Chicago, IL, USA), 2 mM L-glutamine, and 15 µg/mL gentamicin (Gibco, Thermo Fisher Scientific, Waltham, MA, USA). ARPE-19 cells were cultivated at 37 °C in a humidified 5% CO_2_ incubator, tested for mycoplasma contamination, and grown in t-25 flasks.

#### 2.2.2. Enrichment of ARPE-19 Cells with Lipofuscin Granules

To feed the ARPE-19 cell culture with lipofuscin, the cells in suspension were seeded on 35 mm cell culture imaging dishes (MatTek, Ashland, MA, USA) with a concentration of 100,000 cells per dish and cultured there for 2 days. Then, 20 µL of the stock solution of lipofuscin granules (LG) was added to the Petri dish with the cells to achieve a concentration of approx. 300 granules per cell. The control sample was the ARPE-19 cells without the addition of LG. Afterward, cells were cultivated under standard conditions for 24 h.

#### 2.2.3. Photo-Oxidation Protocol

Two groups of ARPE-19 cell samples, consisting of a control sample (ARPE-19 cells without LG) and LG-fed ARPE-19 cells, were used in experiments. The first group of samples was stored in a CO_2_ incubator under standard cultivation conditions in complete darkness. The second group of samples was irradiated for 18 h using a custom-made LED illumination box located inside the CO_2_ incubator. A photograph of the box and the spectrum of the illumination are provided in Supplementary Figure S1. LEDs were mounted at the bottom of the box, where the Petri dish with the cells was located. The illumination light intensity in this area was 0.3 mW/cm^2^.

#### 2.2.4. Cell Viability Assessment Protocol

A cell viability assay was carried out by direct counting of cells (live/dead) based on the laser confocal observations performed in FluoroBrite™ DMEM (Gibco, Thermo Fischer Scientific, Waltham, MA, USA) media. Calculations were made by direct observations, and images were captured and processed using NIS Elements software (Nikon, Tokyo, Japan). Live cells were visualized by fluorescein diacetate (FDA, Thermo Fischer Scientific, Waltham, MA, USA), and dead cells were visualized by nuclei labeling using Sytox Deep Red (Thermo Fischer Scientific, Waltham, MA, USA).

For the MTT test, cells were placed in a 96-well plate (~1500 cells per well) and incubated under standard conditions for 3 days. Lipofuscin loading and irradiation were performed as described in Section 2.2.2 and Section 2.2.3. At 1 and 48 h after the irradiation, 20 μL of 3-(4,5-dimethylthiazole-2-yl)-2,5-diphenyltetrazoliumbromide (MTT) solution (5 mg/mL in PBS) was added, and the samples were incubated at 37 °C for 4 h. The formed formazan crystals were dissolved in DMSO and assessed colorimetrically at 550 nm using an Infinite 200 Pro plate reader (Tecan, Zürich, Switzerland).

The statistical analysis of the data on cell viability was performed by two-way ANOVA with Tukey’s multiple comparison *t*-test using Prism software (GraphPad, Boston, MA, USA).

#### 2.2.5. Production of Recombinant BmCBP Carotenoprotein as a Holoform with Zeaxanthin

The construct of the His_6_-tagged carotenoid-binding domain of *Bombyx mori* carotenoid-binding protein (BmCBP; Uniprot ID: Q8MYA9, residues 68–297) was obtained earlier [52]. Using this genetic construct, the functional BmCBP was reconstituted in zeaxanthin (ZEA)-producing genetically modified C41(DE3) cells, which were pre-transformed with the pACCAR25ΔcrtX plasmid (chloramphenicol resistance) harboring the gene cluster including *crtY*, *crtI*, *crtB*, *crtZ*, and *crtE* sequences from *Erwinia uredovora* allowing for constitutive ZEA production [56]. More details are available in our previous works [49,52,57].

C41(DE3) cells with the overexpressed protein were then lysed, and the BmCBP holoform was purified using a combination of subtractive immobilized metal-affinity and size-exclusion chromatography, followed by hydroxyapatite chromatography for the apoform removal [52]. The resulting product yielded the yellow-colored water solution of BmCBP in a holoform with ZEA (BmCBP-ZEA), which was then sterilized and dissolved in DMEM for further administration to ARPE-19 cells. Special care was taken to ensure that no buffers used during BmCBP purification for the present study contained sodium azide.

#### 2.2.6. Administration of BmCBP Complexed with ZEA to ARPE-19 Cells Fed with Lipofuscin Granules

After incorporation with lipofuscin granules (LG) and prior to irradiation, ARPE-19 cells in a Petri dish with a glass bottom were supplemented with DMEM containing BmCBP-ZEA at 200 nM final concentration and afterwards were maintained in accordance with the cell cultivation protocol described above. According to our previous studies [58], BmCBP-ZEA is stable at temperatures up to 50 °C; hence, it preserves its functionality and carotenoid solubility under conditions of cell cultivation. After the addition of BmCBP-ZEA, cells were maintained according to the protocol of photo-oxidation described above.

### 2.3. Fluorescence Microscopy Measurements

#### 2.3.1. Laser Confocal Microscopy

The laser confocal microscopy imaging was performed using an Eclipse Ti-E microscope with confocal module A1 (Nikon Corporation, Tokyo, Japan) with a 532 nm excitation and detection in the 570–620 nm spectral channel. The cell samples were measured in the chamber under controlled standard cultivation conditions. Data were visualized using Nikon proprietary software.

#### 2.3.2. Fluorescence Lifetime Imaging Microscopy

Fluorescence lifetime imaging (FLIM) was performed in the time-correlated single-photon-counting (TCSPC) mode using the confocal system DCS-120 (Becker&Hickl, Berlin, Germany) installed on the Eclipse Ti2 (Nikon, Tokyo, Japan) microscope. Excitation was performed with a 473 nm picosecond laser BDS-SM-473-LS-101 (Becker&Hickl, Berlin, Germany) with a 30 ps duration impulse driven at 50 MHz repetition rate synchronized with hybrid detector HMP-100-40 (Becker&Hickl, Berlin, Germany) via board SPC-150 (Becker&Hickl, Berlin, Germany). The detection was performed using a 485 nm long-pass filter (Thorlabs, Newton, NJ, USA) or cutting-band filter at 530 nm with 40 nm band width (Thorlabs, Newton, NJ, USA). The choice of the detection window was made according to the spectral characteristics of lipofuscin fluorescence emission at blue light excitation [59].

#### 2.3.3. FLIM Data Analysis

Raw FLIM data were collected using SPCM software and processed via SPCImage (both by Becker&Hickl, Berlin, Germany). The image size was 512 × 512 pixels. In each i-pixel of the image, the fluorescence decay curve $I^{i}\left(t\right)$ was analyzed with triple-exponential approximation using the formula:

(1) $$I^{i}\left(t\right)=A_{1}^{i}e^{-\frac{t}{\tau_{1}^{i}}}+A_{2}^{i}e^{-\frac{t}{\tau_{2}^{i}}}+A_{3}^{i}e^{-\frac{t}{\tau_{3}^{i}}},$$

where $\tau_{1}^{i},\tau_{2}^{i},\tau_{3}^{i}$—lifetimes and $A_{1}^{i},A_{2}^{i},A_{3}^{i}$—amplitudes of the components of the fluorescence decay curve in i-pixel. In each i-pixel, the amplitude-weighted average fluorescence lifetime was calculated as follows:

(2) $$\tau_{av}^{i}=\frac{A_{1}^{i}\tau_{1}^{i}+A_{2}^{i}\tau_{2}^{i}+A_{3}^{i}\tau_{3}^{i}}{A_{1}^{i}+A_{2}^{i}+A_{3}^{i}}.$$

In total, eight sets of parameters were obtained: {$\tau_{av}$},{$\tau_{1}$},{$\tau_{2}$},{$\tau_{3}$},{$A_{1}$},{$A_{2}$},{$A_{3}$}, and {$\chi^{2}$}, where $\chi^{2}$ is a parameter of the quality of the fluorescence decay fitting using triple-exponential decay function. Each set contained 262,144 values. These sets were visualized as distributions and processed in Origin 2018 software (OriginLab Corporation, Northampton, MA, USA) by fitting with the Gauss function. Average fluorescence lifetime (<$\tau_{av}$>), average values of lifetime of each component (<$\tau_{1}$>, <$\tau_{2}$>, <$\tau_{3}$>), average values of each component amplitude (<$A_{1}$>, <$A_{2}$>, <$A_{3}$>), and average <$\chi^{2}$> were obtained as the central point of the Gaussian; errors of the average values correspond to the full width at half magnitude of the Gaussian. Each experiment included N = 3 different images. The described procedure was applied independently to each image.

To characterize the results of the whole experiment considering the data of each image, the mean values were calculated:

(3) $${\bar{\tau }}_{av}=\frac{\sum_{j=1}^{N=3}\langle \tau_{av}^{j}\rangle }{3};\;{\bar{\tau }}_{1}=\frac{\sum_{j=1}^{N=3}\langle \tau_{1}^{j}\rangle }{3};\;{\bar{A}}_{1}=\frac{\sum_{j=1}^{N=3}\langle A_{1}^{j}\rangle }{3};\ldots \;\bar{\chi^{2}}=\frac{\sum_{j=1}^{N=3}\langle \chi^{2}{}^{j}\rangle }{3},$$

where *N* = 3 is the number of obtained images. Errors in the calculations of the mean values correspond to standard deviations.

## 3. Results

### 3.1. Characteristics of Lipofuscin Granules-Fed RPE Cell Samples

**Figure 1:**
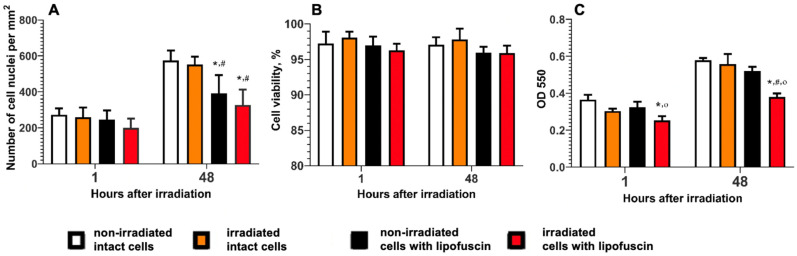
Assessment of the cyto- and phototoxic effects of lipofuscin granules loaded into ARPE-19 cells, depending on the time of incubation after white light irradiation: (**A**) the number of cells per mm^2^ (*n* = 5); (**B**) results of live/dead test (*n* = 5); (**C**) results of MTT assay (*n* = 3). The data were analyzed by two-way ANOVA with Tukey’s multiple comparison *t*-test and represented as means ± standard deviations. * Differences in comparison with the non-irradiated intact (without lipofuscin) cells (control group); # differences in comparison with the irradiated intact cells; ^ο^ differences in comparison with non-irradiated cells loaded with lipofuscin.

First, we performed tests of the viability of LG-fed ARPE-19 cells. The results are presented in Figure 1. Figure 1A demonstrates how the number of ARPE-19 cells changed in the dark and under light exposure depending on the time of incubation. It is seen that in case of a short time period (1 h) after the irradiation was finished (Figure 1A, left set of diagrams), the number of living cells did not change significantly in different cell samples, with a slightly decreasing trend in the sample of irradiated LG-fed cells. This can be explained by the fact that this specific sample was exposed to two external factors: white light irradiation and lipofuscin incorporation, both of which are capable of causing cytotoxic effects. However, photo-oxidation and the oxidative stress it induces lead to a number of effects developing over time, affecting cell division. This was confirmed when the cell number was measured at 48 h after the irradiation was finished (Figure 1A, right set of diagrams): we can see that the decreasing trend in the rate of the cell’s division was more pronounced in cases when external influences were applied. While the effect of white light irradiation was not significant, the cytotoxic effect of lipofuscin itself was strong even without the irradiation, decreasing the cell culture growth rate. It is clearly seen that the irradiation additionally enhanced the stand-alone toxic effect of lipofuscin observed in the dark, and the decrease in the number of living cells was the most significant after 48 h compared to the control group. Meanwhile, the cell’s viability was stable in the range of 95–98% under the application of any external stimuli (Figure 1B). In fact, the incorporation of lipofuscin into ARPE-19 cells did decrease the cell viability; however, the effect was not statistically significant. The results of the MTT test (Figure 1C) demonstrated that the optical density (OD), related to the concentration of formazan formed due to the cell metabolic activity, significantly decreased during the incubation of the ARPE-19 cells loaded with lipofuscin, both in the early stages and two days after irradiation, compared with the control group of intact cells. Since the OD of the solution depends not only on the cell number or proliferation but also on the mitochondrial respiration, the obtained results can indirectly point to changes in the cellular energy capacity. Taking all the results into account, it can be concluded that the observed effect was associated with the inhibition of cellular metabolism but not with a change in the number of cells. This agrees with the results of the studies conducted by other groups [60,61] and with our previously obtained results [62]. It should be noted that non-irradiated cells treated with lipofuscin showed a slight MTT signal decrease only after 48 h of incubation. Overall, it allows us to conclude that general supportive intracellular molecular mechanisms did not lose their effectiveness completely with the incorporation of lipofuscin, even during the prolonged incubation. In other words, even in 48 h after the white light irradiation finished, the LG-fed ARPE-19 cells mostly preserved their molecular machinery to counteract the external aggressive factors of lipofuscin and irradiation, and hence might be considered as an adequate experimental model.

### 3.2. Intracellular Localization of Lipofuscin Granules in ARPE-19 Cells

**Figure 2:**
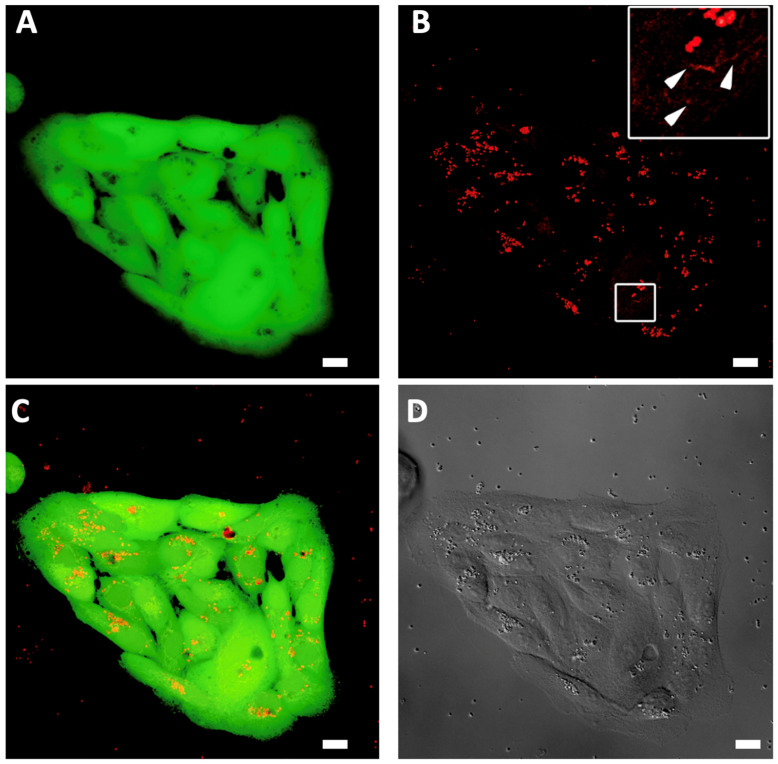
Confocal microscopy imaging of ARPE-19 cells fed with lipofuscin granules and incubated for 48 h after white light irradiation. Excitation: 532 nm. (**A**) living cells labeled by fluorescein diacetate (FDA); (**B**) lipofuscin granules autofluorescence in 570–620 nm detection channel (white arrows point to perinuclear areas with structure relevant to mitochondria morphology); (**C**) merged; (**D**) transmitted light channel. The measuring bar corresponds to 10 μm.

Since a long 48 h incubation of LG-fed ARPE-19 cells after exposure to white light demonstrated a significant decrease in the cell number, we visualized the intracellular localization of lipofuscin granules and characterized the mechanisms of its cytotoxic manifestations in our experimental model by laser scanning confocal microscopy. Cell images are presented in Figure 2. According to Figure 2B, upon endocytosis, LG tend to localize in the perinuclear areas (pointed out with white arrows in Figure 2B) and, therefore, possibly interfere with the mitochondria. This partially explains the mechanisms of the lipofuscin-associated cytotoxic effect observed in the absence of the irradiation. When loaded into cells, LG might be localized in proximity to mitochondria and damage the mitochondrial membranes, disrupting the electron transport chains. A similar effect was observed in skeletal muscle cells, where the age-related cytotoxic effect of lipofuscin was comparable to the action of electron transfer chain inhibitors leading to the massive formation of ROS and free radicals [63].

### 3.3. Fluorescence Lifetime Imaging (FLIM) of Lipofuscin Granules-Fed RPE Cells

**Figure 3:**
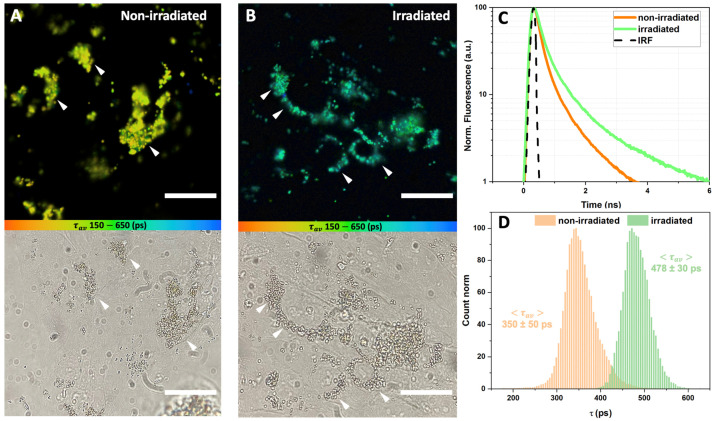
FLIM data on ARPE-19 cells fed with lipofuscin granules, measured immediately after exposure to white light. Excitation λexc: 473 nm; detection λdet: 485 (long pass) nm; color scale encodes the range of average lifetimes from 150 ps (orange) to 650 ps (blue). (**A**) FLIM and transmitted microscopy images of non-irradiated cells; (**B**) FLIM and transmitted microscopy images of irradiated cells; (**C**) fluorescence decay curves averaged for the set of the curves calculated in each pixel of the corresponding image; (**D**) distribution of the fluorescence lifetime in each pixel of the image. Average fluorescence lifetime values ($<\tau_{av}>$) were obtained by approximation of distributions with the Gauss function, with errors corresponding to the width of the Gaussian. White arrows correspond to the deposition of LG in ARPE-19 cells. The measuring bar corresponds to 10 μm.

**Figure 4:**
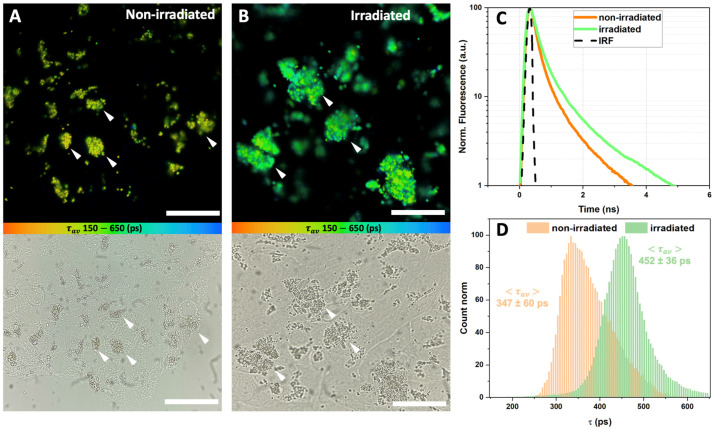
FLIM data on ARPE-19 cells fed with lipofuscin granules, measured at 48 h after white light irradiation. Excitation λexc: 473 nm; detection λdet: 485 (long pass) nm; color scale encodes the range of average lifetimes from 150 ps (orange) to 650 ps (blue). (**A**) FLIM and transmitted microscopy images of non-irradiated cells; (**B**) FLIM and transmitted microscopy images of irradiated cells; (**C**) fluorescence decay curves, averaged on the set of the curves calculated in each pixel of the corresponding image; (**D**) distribution of fluorescence lifetime in each pixel of the image. Average fluorescence lifetime values ($<\tau_{av}>$) were obtained by approximation of distributions with the Gauss function with errors corresponding to the width of the Gaussian. White arrows correspond to the deposition of lipofuscin granules in RPE cells. The measuring bar corresponds to 10 μm.

The results of the FLIM of the LG, phagocytized by ARPE-19 cells, are presented in Figure 3 (FLIM was performed immediately after irradiation) and Figure 4 (FLIM was performed at 48 h after irradiation). It can be seen that the LG are accumulated in the cell cytoplasm, predominantly in the perinuclear zone, during the preliminary incubation. Figure 3C and Figure 4C show the average fluorescence decay curves of lipofuscin, and Figure 3D and Figure 4D represent the distributions of the amplitude-weighted average lifetime values of lipofuscin fluorescence obtained from each pixel of the FLIM image.

When FLIM was performed right after the irradiation, the fluorescence lifetime distribution of either non-irradiated or irradiated lipofuscin was characterized with homogeneous single-modal straight narrow peaks (Figure 3D). Approximation with the Gauss function meant that the non-irradiated-state lipofuscin’s average fluorescence lifetime was $<\tau_{av}>$ = 350 ± 50 ps. When cells with lipofuscin were white-light-irradiated, one can observe a significant increase in the average lifetime of lipofuscin fluorescence ($<\tau_{av}>$ = 478 ± 30 ps). Analysis of the fluorescence decay data obtained in three different independent measurements (images) revealed a shift in each of the three components (Table 1). The increase in the fast time component $\tau_{1}$ was approximately 34% after white light exposure, in comparison with intact non-irradiated lipofuscin. Meanwhile, the changes in a longer time component $\tau_{2}$ were even more pronounced, and the relative increase was 40%. The irradiation-caused increase in the longest component $\tau_{3}$ was less pronounced but still significant (ca. 18%). The average amplitudes of each component of the lipofuscin fluorescence profile did not change significantly after irradiation, indicating that photoinduced changes in lipofuscin fluorescence are related to the changes in chromophore state and the corresponding quantum yield but not to the relative concentration of the states. In every image, $\chi^{2}$ was close to 1.0, which implies the good quality of the multiexponential approximation.

Similar results were observed when FLIM was performed after 48 h of incubation after LG-fed cells were irradiated (Figure 4). In the case of the non-irradiated lipofuscin, the fluorescence lifetime $<\tau_{av}>$ was 347 ± 60 ps, and in the case of the irradiated lipofuscin it increased to 452 ± 36 ps (Figure 4D). The increase in $\tau_{av}$ can be observed in Figure 4D, and despite being slightly less pronounced in comparison with the experiment when FLIM was performed right after the irradiation (Figure 3D), it is still significant. According to the analysis of the fluorescence decay data, obtained in three independent measurements, a 12% increase in the fast time component $\tau_{1}$ was observed, while the increase in the longer components $\tau_{2}$ and $\tau_{3}$ was less pronounced and estimated at ca. 1–3% (Table 1). At the same time, in contrast to the previous experiment, we observed a slight redistribution of the amplitudes of the components: in the non-irradiated sample, the mean amplitude of the fast component $A_{1}$ predominated; its average value was 87–88%, and after the irradiation it decreased to 80–81%. Meanwhile, at the expense of the decrease in $A_{1}$, the irradiation led to an increase in $A_{2}$ from 10% (non-irradiated sample) to 17% (irradiated sample), and an increase in $A_{3}$ from 1.5% to 3.2%. We suggest that these observations correspond to changes in the lipofuscin chromophore composition caused by the long-term effects of oxidative stress.

### 3.4. Effect of the Carotenoprotein-Mediated Carotenoid Delivery on the Photo-Oxidation of Lipofuscin in ARPE-19 Cells

**Figure 5:**
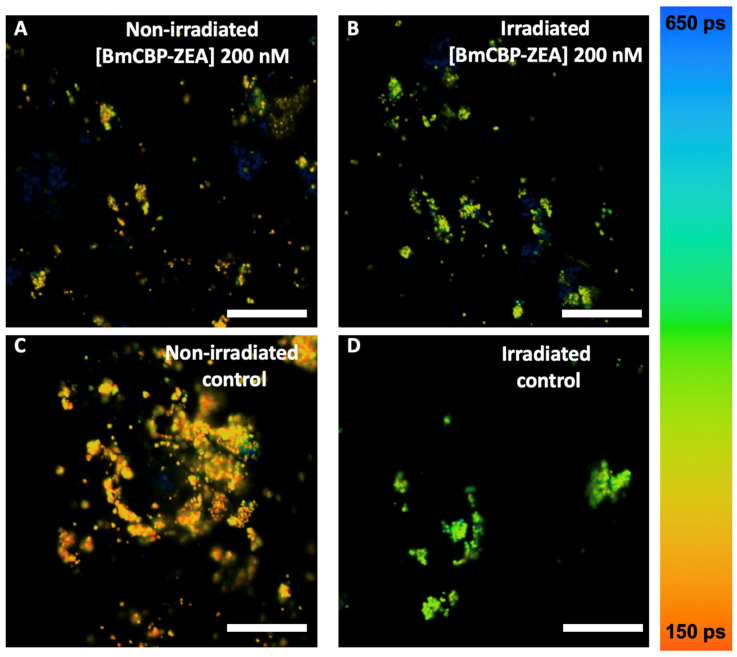
Effect of BmCBP-ZEA on the lifetime of fluorescence of lipofuscin granules, phagocytized by ARPE-19 cells, after irradiation by white light for 18 h. FLIM parameters: excitation λexc: 473 nm; detection λdet: 530(40) nm; color scale encodes the range of lifetimes from 150 ps (orange) to 650 ps (blue). (**A**,**B**) Prior to the irradiation, cells were supplemented with media containing 200 nM BmCBP-ZEA. (**C**,**D**) Cell samples without BmCBP-ZEA (control). Measurements were performed immediately after irradiation finished. The measuring bar corresponds to 10 μm.

To assess the efficiency of the ZEA delivery into the RPE cells by the BmCBP carotenoprotein, we performed an experiment to investigate how its addition to lipofuscin-fed ARPE-19 cells affects the lipofuscin fluorescence lifetime. Figure 5 represents FLIM data obtained immediately after irradiation. Firstly, white light irradiation led to a significant increase in lipofuscin fluorescence lifetime in comparison with non-irradiated lipofuscin (Figure 5D vs. Figure 5C). This corresponds with the results described in the previous section with the average lifetimes of the lipofuscin fluorescence being slightly shorter because of the different detection windows. Secondly, in the samples supplemented with BmCBP-ZEA, irradiation did not change the lifetime of the lipofuscin fluorescence as much drastically (Figure 5A vs. Figure 5B).

**Figure 6:**
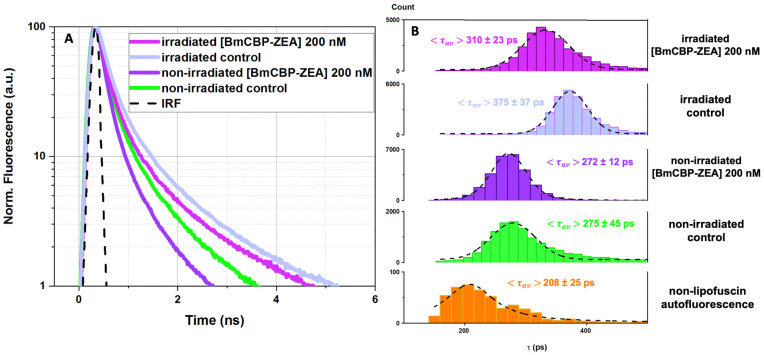
Effect of BmCBP-ZEA on the changes in lipofuscin fluorescence lifetime induced by photo-oxidation with white light irradiation: (**A**) average fluorescence decay curves; (**B**) distribution of fluorescence lifetime in each pixel of the image. Excitation λexc: 473 nm; detection λdet: 530(40) nm. Average fluorescence lifetime values ($<\tau_{av}>$) were obtained by approximation of distributions with the Gauss function (fitting is visualized as dashed line) with errors corresponding to the width of the Gaussian.

Figure 6 provides the distributions of the average fluorescence lifetime for irradiated and non-irradiated LG-fed ARPE-19 cells with and without the addition of BmCBP-ZEA. Figure 6A shows the fluorescence decay curves. One can see that the decay curve of the lipofuscin fluorescence in irradiated cells supplemented with BmCBP-ZEA is different in comparison with the decay curve of the fluorescence of lipofuscin in cells without BmCBP-ZEA. Figure 6B shows the distributions of the average lifetime values. The distributions were fitted using the Gauss function. One can see that, after white light irradiation, the presence of BmCBP-ZEA suppressed the increase in the mean fluorescence lifetime. After irradiation, in cells supplemented with BmCBP-ZEA, $<\tau_{av}>$ = 310 ± 23 ps (non-irradiated control, $<\tau_{av}>$ = 272 ± 12 ps) and in the irradiated cells without BmCBP-ZEA, $<\tau_{av}>$ = 375 ± 37 ps (non-irradiated control, $<\tau_{av}>$ = 275 ± 45 ps). A comparison of the obtained values indicates an approx. 35% inhibition of the photoinduced oxidation at a given concentration of BmCBP-ZEA.

## 4. Discussion

The cytotoxic mechanism of lipofuscin photo-oxidation includes both a direct interaction between an activated sensitizer and the cell components, and the indirect formation of ROS, which, in turn, interact and damage cellular nucleic acids, proteins, and lipids [62]. It is known that the toxic singlet oxygen formed after A2E (lipofuscin component) irradiation with blue light not only damages the biochemical components of the cell but also affects the A2E molecules themselves, converting them into an even more toxic form [64]. Lipofuscin granules have bright fluorescence compared to the background autofluorescence of RPE cells, which is 1000 times lower than the fluorescence intensity of lipofuscin (see Supplementary Figure S2), making it easy to visualize with conventional fluorescence microscopy. However, this approach does not allow assessing the functional state of lipofuscin and evaluating its hazards to the cell. In this study, we attempted to solve this problem and demonstrated that the exposure of lipofuscin granules in ARPE-19 cells to white light leads to a significant increase in lipofuscin fluorescence lifetime (Figure 3 and Figure 4), which is very likely an indicator of pathological changes and possible negative consequences for the cell culture (Figure 1).

In contrast to the fluorescence intensity, the lifetime does not depend on the concentration and directly reflects changes in the quantum yield of fluorescence, which is determined by the functional properties of the fluorophore [65]. Thus, FLIM is a promising approach for obtaining functional images of tissues containing lipofuscin to assess their condition and identify pathologies. The probability of the ROS formation is directly proportional to the concentration of oxygen and photosensitizer molecules in the excited state. Therefore, it is obvious that photosensitizers with a high quantum yield and, consequently, higher lifetime of the excited state are the most dangerous for the cell under aerobic conditions. Accordingly, to reduce the probability of generating ROS, a common strategy is to reduce the lifetime of the excited molecule [66,67]. This strategy is well developed by photosynthetic organisms, which utilize carotenoids for protection against photodamage. Carotenoids, due to the very short lifetimes of their excited states (typically ~100–200 fs for S2 state and ~3–10 ps for S1 [68]), can quickly and efficiently convert the energy of electron excitation into heat. This is also relevant for the energy absorbed by other molecules in the local environment of the carotenoid.

Having tested the method of functional imaging of lipofuscin with FLIM on ARPE-19 cells, we examined the effects of the natural antioxidant zeaxanthin (ZEA) delivered by the water-soluble carotenoprotein BmCBP to pigment epithelium cells containing lipofuscin granules with and without exposure to white-light-inducing photo-oxidation. Our experiments show that the addition of the carotenoprotein to the culture medium leads to changes in the fluorescence lifetime distributions of lipofuscin: when BmCBP-ZEA was added, the photoinduced increase in the lipofuscin average lifetime was significantly suppressed (Figure 6). This observation suggests that the photoinducible oxidative stress is less pronounced when the protein delivers the carotenoid into the cell membrane. In a previous study [54], we demonstrated that the apoforms of BmCBP (protein not complexed with carotenoid) did not have any effects on the model cell culture, and, moreover, it is likely that the protein is unable to penetrate the cell membrane, similar to other carotenoproteins [52]. Thus, BmCBP acts as an effective transport device for ZEA delivery into the membrane, which is important for its wide application in industry.

**Figure 7:**
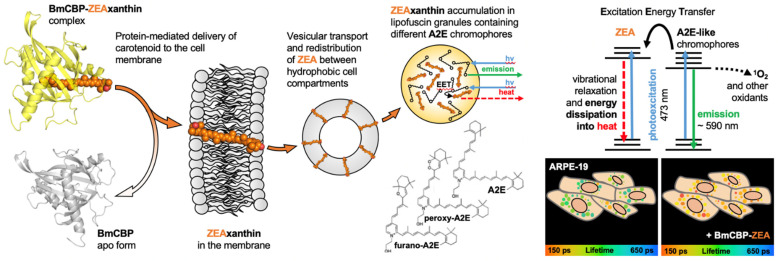
Schematic representation of the effects of protein-mediated delivery of zeaxanthin to ARPE-19 cells containing lipofuscin granules. The structure of water-soluble BmCBP (yellow cartoon, according to PDB ID 7ZVR) allows it to effectively bind zeaxanthin (ZEA, shown as orange spheres). When it contacts ARPE-19 cell membranes, the carotenoid is transferred into the membrane and an apoform of the protein (grey cartoon, according to PDB ID 7ZTQ) is formed. Further, probably through basic cellular transport mechanisms, carotenoids can be redistributed between the membranes of various cell organoids including mitochondria. Due to their high hydrophobicity and a certain similarity of chemical structures of carotenoids and A2E-like chromophores, carotenoids can be transported into lipofuscin granules. The close proximity of zeaxanthin and the main lipofuscin fluorophores (A2E) can lead to an energy coupling that results in a reduction in the fluorescence quantum yield of the lipofuscin fluorophores detected by FLIM. Energy transfer to zeaxanthin, as well as its direct photoexcitation, leads to rapid energy dissipation, which ultimately reduces the probability of generation of reactive oxygen species and suppresses the oxidative stress.

The subsequent redistribution of the carotenoid throughout the cell upon protein-mediated delivery is probably carried out by endocytosis and vesicular transport mechanisms [69]. We have previously confirmed carotenoid presence in different subcellular structures by Raman microscopy of the cells after echinenone delivery by AnaCTDH carotenoprotein [53]. As a result, the carotenoid may end up in various hydrophobic compartments of the cell and probably in lipofuscin granules as well. This scenario was confirmed by the significant decrease in the duration of the excited states of lipofuscin fluorophores observed in the experiment (Figure 6) as a result of zeaxanthin delivery. Figure 7 shows possible interactions between zeaxanthin and lipofuscin fluorophores, explaining the decrease in the lifetime of the A2E-like chromophores.

Given the overlap of the fluorescence spectrum of lipofuscin and the carotenoid absorption, we assume that the electronic excitation energy of lipofuscin can be transferred to the carotenoid and, as a result, converted into heat due to vibrational relaxation. We speculate that the multichromophore structure of the lipofuscin granule promotes delocalization of electron excitation between neighboring chromophores, and the multistep energy transfer leads to the possibility of a single quencher molecule capturing excitation. Thus, this constitutes the most likely mechanism of the shortening of lipofuscin lifetime driven by carotenoid delivery. The subsequent irradiation of cells containing lipofuscin granules with carotenoid leads to an increase in the average lifetime of fluorescence, but in this case the effect does not exceed the level of the control sample before irradiation (Figure 6). As the duration of the excited states of lipofuscin remains low, we can postulate that the carotenoid prevents the further photo-oxidation of lipofuscin and consequent cytotoxic effects. However, changes in the lifetime of lipofuscin fluorescence cannot provide an exact number of the delivered carotenoid molecules, since energy transfers may occur between multiple molecules. In order to measure the intracellular concentration of carotenoids upon the delivery, one would need to track carotenoids during their intracellular transportation. The implementation of fluorescence imaging is limited in this case because the conjugation of the carotenoid with a fluorescent label may interfere with its binding by the carotenoprotein.

Our study also demonstrated that the FLIM of lipofuscin granules can be utilized as an additional tool in advance fluorescence diagnostics of retinal pathologies characterized by lipofuscin accumulation. Previous experimental research demonstrated that fundus autofluorescence (FAF) imaging based on the probing of lipofuscin granule fluorescence quantitative and qualitative properties was an effective approach for the diagnosis of the pathological development of the RPE [55,70,71,72]. According to the results of an FAF spectrometric study in vivo [73], the presence of lipofuscin can be observed in the RPE of healthy eyes without pathologies; however, as individuals grow older, the lipofuscin fluorescence significantly increases, which points to the age-related increases in lipofuscin concentration. Further studies demonstrated that, in addition to the integral autofluorescence intensity of lipofuscin used as a diagnostic parameter, the spectral characteristics of lipofuscin autofluorescence can also provide useful diagnostic information [74]. Nowadays, a lot of research is underway to expand the capabilities of the fluorescence diagnostic method to include the time-resolved measurements of lipofuscin fluorescence as a part of fluorescence lifetime imaging ophthalmoscopy (FLIO). This technique has already been implemented in some pilot in vivo studies and demonstrated promising results. For instance, healthy eyes have been shown to exhibit different lifetime patterns of lipofuscin fluorescence than those of eyes with age-related macular degeneration [75]. Specific changes in the fluorescence lifetime of lipofuscin have also been detected for retinitis pigmentosa [76], Stargardt disease [77], and macular telangiectasia type 2 [78], and in the eyes of patients with diabetes [79] and Alzheimer’s disease [80]. Our results, presented in the current study, revealed that fluorescence lifetime imaging of lipofuscin is sensitive to oxidative stress which is exhibited in the retinal pigment epithelium. The lipofuscin fluorescence lifetime significantly increased in our in vitro experimental model involving white light irradiation-induced photo-oxidation. Therefore, it can be proposed as a novel bio-optical indicator of the oxidative stress occurring in the eye tissue.

## 5. Conclusions

In this study, we demonstrated that the fluorescence lifetime of lipofuscin granules, phagocytized by ARPE-19 cells, increased after white-light-induced photo-oxidation. Using time-resolved fluorescence imaging (FLIM), we observed that protein-mediated zeaxanthin delivery had an effect on the state of lipofuscin by reducing its pigment excited-state lifetime. We propose that carotenoids delivered into the cell can not only shield lipofuscin, preventing its excitation and interaction with the products of its oxidation, but also act as quenchers of excited states due to the energy coupling between the molecules.

The recent advances in the use of genetically modified *E. coli* strains allows the reconstitution of water-soluble carotenoproteins [49,52,53,57] in the amounts necessary to fulfill the demand for antioxidants in different markets, including cosmetics, pharmaceuticals, and food biotechnology. Lipofuscin accumulates in various cells in the human organism. The fundamental solubility of selected carotenoproteins in aqueous media allows antioxidants to be delivered to different tissues. Thus, we present new experimental possibilities for the diagnostics of oxidative stress and approaches to modulate it using protein-mediated delivery of antioxidants.

## Figures and Tables

**Table 1:** Results of the analysis of FLIM images of lipofuscin-granule-fed ARPE-19 cells with and without photo-oxidation under white light irradiation. Results were obtained by the analysis of N = 3 different independent experiments. Data correspond to mean values; errors represent standard deviations. Excitation λexc: 473 nm; detection λdet: 485 (long pass) nm.

Sample	$\bar{\tau_{av}}\;(\mathrm{ps})$	$\bar{\tau_{1}}\;\;(\mathrm{ps})$	$\bar{A_{1}}\;(\%)$	$\bar{\tau_{2}}\;\;(\mathrm{ps})$	$\bar{A_{2}}\;(\%)$	$\bar{\tau_{3}}\;\;(\mathrm{ps})$	$\bar{A_{3}}\;(\%)$	$\bar{\chi^{2}}$
0 h After Irradiation								
Non-irradiated	323 ± 23	243 ± 15	83.8 ± 0.9	487 ± 73	14.1 ± 0.6	2109 ± 87	2.1 ± 0.4	0.893
Irradiated	482 ± 10	368 ± 51	83.2 ± 0.4	817 ± 90	14.5 ± 0.2	2564 ± 95	2.3 ± 0.6	0.832
48 h After Irradiation								
Non-irradiated	311 ± 17	224 ± 21	87.8 ± 1.0	538 ± 37	10.7 ± 0.9	2348 ± 84	1.5 ± 0.1	0.880
Irradiated	439 ± 16	253 ± 20	80.5 ± 2.1	554 ± 81	17.1 ± 1.9	2355 ± 145	3.3 ± 0.5	0.861

## Data Availability

All the necessary data are provided in the figures in the present paper and in its Supplementary Materials.

## Supplementary Materials

The following supporting information can be downloaded at: https://www.mdpi.com/article/10.3390/antiox12020413/s1, Supplementary Figure S1: A—photograph of the diode light lamp inside the plastic container, which was put into the CO_2_ incubator; B—spectrum of the diode lamp light, which was used in the experiments. Supplementary Figure S2: Autofluorescence FLIM images of intact ARPE-19 cells: A—non-irradiated; B—irradiated with white diode lamp light. Brightness and contrast greatly (1000 times) increased.

## References

[1] Geng L., Wihlmark U., Algvere P.V. (1999). Lipofuscin Accumulation in Iris Pigment Epithelial Cells Exposed to Photoreceptor Outer Segments. Exp. Eye Res..

[2] Boulton M., Dontsov A., Jarvis-Evans J., Ostrovsky M., Svistunenko D. (1993). Lipofuscin Is a Photoinducible Free Radical Generator. J. Photochem. Photobiol. B Biol.

[3] Yakovleva M., Dontsov A., Trofimova N., Sakina N., Kononikhin A., Aybush A., Gulin A., Feldman T., Ostrovsky M. (2022). Lipofuscin Granule Bisretinoid Oxidation in the Human Retinal Pigment Epithelium Forms Cytotoxic Carbonyls. Int. J. Mol. Sci..

[4] Pan C., Banerjee K., Lehmann G.L., Almeida D., Hajjar K.A., Benedicto I., Jiang Z., Radu R.A., Thompson D.H., Rodriguez-Boulan E. (2021). Lipofuscin Causes Atypical Necroptosis through Lysosomal Membrane Permeabilization. Proc. Natl. Acad. Sci. USA.

[5] Davies S., Elliott M.H., Floor E., George Truscott T., Zareba M., Sarna T., Shamsi F.A., Boulton M.E. (2001). Photocytotoxicity of Lipofuscin in Human Retinal Pigment Epithelial Cells. Free Radic. Biol. Med..

[6] Sparrow J.R., Duncker T. (2014). Fundus Autofluorescence and RPE Lipofuscin in Are-Related Macular Degeneration. J. Clin. Med..

[7] Kathleen Dorey C., Wu G., Ebensrein D., Garsd A. (1989). Cell Loss in the Aging Retina Relationship to Lipofuscin Accumulation and Macular Degeneration. Investig. Ophthalmol. Vis. Sci..

[8] Lu L.J., Liu J., Adelman R.A. (2017). Novel Therapeutics for Stargardt Disease. Graefe’s Arch. Clin. Exp. Ophthalmol..

[9] Kennedy C.J., Rakoczy P.E., Constable I.J. (1995). Lipofuscin of the Retinal Pigment Epithelium: A Review. Eye.

[10] Schutt F., Ueberle B., Schnölzer M., Holz F.G., Kopitz J. (2002). Proteome Analysis of Lipofuscin in Human Retinal Pigment Epithelial Cells. FEBS Lett..

[11] Ng K.P., Gugiu B., Renganathan K., Davies M.W., Gu X., Crabb J.S., Kim S.R., Rózanowska M.B., Bonilha V.L., Rayborn M.E. (2008). Retinal Pigment Epithelium Lipofuscin Proteomics. Mol. Cell. Proteom..

[12] Sparrow J.R., Wu Y., Kim C.Y., Zhou J. (2010). Phospholipid Meets All-Trans-Retinal: The Making of RPE Bisretinoids. J. Lipid Res..

[13] Sakai N., Decatur J., Nakanishi K., Eldred G.E. (1996). Ocular Age Pigment “A2E”: An Unprecedented Pyridinium Bisretinoid. J. Am. Chem. Soc..

[14] Ben-Shabat S., Parish C.A., Vollmer H.R., Itagaki Y., Fishkin N., Nakanishi K., Sparrow J.R. (2002). Biosynthetic Studies of A2E, a Major Fluorophore of Retinal Pigment Epithelial Lipofucin. J. Biol. Chem..

[15] Sparrow J.R., Fishkin N., Zhou J., Cai B., Jang Y.P., Krane S., Itagaki Y., Nakanishi K. (2003). A2E, a Byproduct of the Visual Cycle. Vis. Res..

[16] Wielgus A.R., Chignell C.F., Ceger P., Roberts J.E. (2010). Comparison of A2E Cytotoxicity and Phototoxicity with All-Trans-Retinal in Human Retinal Pigment Epithelial Cells. Photochem. Photobiol..

[17] Różanowska M., Wessels J., Boulton M., Burke J.M., Rodgers M.A.J., George Truscott T., Sarna T. (1998). Blue Light-Induced Singlet Oxygen Generation by Retinal Lipofuscin in Non-Polar Media. Free Radic. Biol. Med..

[18] Dontsov A.E., Glickman R.D., Ostrovsky M.A. (1999). Retinal Pigment Epithelium Pigment Granules Stimulate the Photo-Oxidation of Unsaturated Fatty Acids. Free Radic. Biol. Med..

[19] Ruan Y., Jiang S., Gericke A. (2021). Age-Related Macular Degeneration: Role of Oxidative Stress and Blood Vessels. Int. J. Mol. Sci..

[20] Wihlmark U., Wrigstad A., Roberg K., Erik Nilsson S.G., Brunk U.T. (1997). Lipofuscin Accumulation in Cultered Retinal Pigment Epithelial Cells Causes Enhanced Sensitivity to Blue Light Irradiation. Free Radic. Biol. Med..

[21] Sparrow J.R., Nakanishi K., Parish C.A. (2000). The Lipofuscin Fluorophore A2E Mediates Blue Light-Induced Damage to Retinal Pigmented Epithelial Cells. Investig. Ophthalmol. Vis. Sci..

[22] Sundelin S.P., Nilsson S.E.G. (2001). Lipofuscin-Formation in Retinal Pigment Epithelial Cells Is Reduced by Antioxidants. Free Radic. Biol. Med..

[23] Jang Y.P., Zhou J., Nakanishi K., Sparrow J.R. (2005). Anthocyanins Protect Against A2E Photooxidation and Membrane Permeabilization in Retinal Pigment Epithelial Cells. Photochem. Photobiol..

[24] Khoo H.E., Ng H.S., Yap W.S., Goh H.J.H., Yim H.S. (2019). Nutrients for Prevention of Macular Degeneration and Eye-Related Diseases. Antioxidants.

[25] Sparrow J.R., Vollmer-Snarr H.R., Zhou J., Jang Y.P., Jockusch S., Itagaki Y., Nakanishi K. (2003). A2E-Epoxides Damage DNA in Retinal Pigment Epithelial Cells. Vitamin E and Other Antioxidants Inhibit A2E-Epoxide Formation. J. Biol. Chem..

[26] Alaimo A., di Santo M.C., Domínguez Rubio A.P., Chaufan G., García Liñares G., Pérez O.E. (2020). Toxic Effects of A2E in Human ARPE-19 Cells Were Prevented by Resveratrol: A Potential Nutritional Bioactive for Age-Related Macular Degeneration Treatment. Arch. Toxicol..

[27] Mrowicka M., Mrowicki J., Kucharska E., Majsterek I. (2022). Lutein and Zeaxanthin and Their Roles in Age-Related Macular Degeneration—Neurodegenerative Disease. Nutrients.

[28] Rózanowski B., Cuenco J., Davies S., Shamsi F.A., Zadło A., Dayhaw-Barker P., Rózanowska M., Sarna T., Boulton M.E. (2008). The Phototoxicity of Aged Human Retinal Melanosomes. Photochem. Photobiol..

[29] Li B., Ahmed F., Bernstein P.S. (2010). Studies on the Singlet Oxygen Scavenging Mechanism of Human Macular Pigment. Arch. Biochem. Biophys..

[30] Kim S.R., Nakanishi K., Itagaki Y., Sparrow J.R. (2006). Photooxidation of A2-PE, a Photoreceptor Outer Segment Fluorophore, and Protection by Lutein and Zeaxanthin. Exp. Eye Res..

[31] Roberts J.E., Dennison J. (2015). The Photobiology of Lutein and Zeaxanthin in the Eye. J. Ophthalmol..

[32] Olchawa M.M., Furso J.A., Szewczyk G.M., Sarna T.J. (2017). Lipofuscin-Mediated Photic Stress Inhibits Phagocytic Activity of ARPE-19 Cells; Effect of Donors’ Age and Antioxidants. Free Radic. Res..

[33] Arunkumar R., Gorusupudi A., Li B., Blount J.D., Nwagbo U., Kim H.J., Sparrow J.R., Bernstein P.S. (2021). Lutein and Zeaxanthin Reduce A2E and Iso-A2E Levels and Improve Visual Performance in Abca4−/−/Bco2−/− Double Knockout Mice. Exp. Eye Res..

[34] Sahin K., Gencoglu H., Akdemir F., Orhan C., Tuzcu M., Sahin N., Yilmaz I., Juturu V. (2019). Lutein and Zeaxanthin Isomers May Attenuate Photo-Oxidative Retinal Damage via Modulation of G Protein-Coupled Receptors and Growth Factors in Rats. Biochem. Biophys. Res. Commun..

[35] José R., Torres A., Correa C.R. (2022). The Role of Non-Enzymatic Antioxidants on Age-Related Macular Degeneration. Front. Drug CHemistry Clin. Res..

[36] Lornejad-Schäfer M.R., Lambert C., Breithaupt D.E., Biesalski H.K., Frank J. (2007). Solubility, Uptake and Biocompatibility of Lutein and Zeaxanthin Delivered to Cultured Human Retinal Pigment Epithelial Cells in Tween40 Micelles. Eur. J. Nutr..

[37] Ibrahim A.E., Shafaa M.W., Khedr M.H., Rashed R.F. (2019). Comparative Study between Lutein and Its Liposomal Form on Cisplatin-Induced Retinal Injury in Rabbits. Cutan. Ocul. Toxicol..

[38] Algan A.H., Gungor-Ak A., Karatas A. (2022). Nanoscale Delivery Systems of Lutein: An Updated Review from a Pharmaceutical Perspective. Pharmaceutics.

[39] Ma Y., You T., Wang J., Jiang Y., Niu J. (2022). Research Progress on Construction of Lutein-Loaded Nano Delivery System and Their Improvements on the Bioactivity. Coatings.

[40] Tan C., Xue J., Lou X., Abbas S., Guan Y., Feng B., Zhang X., Xia S. (2014). Liposomes as Delivery Systems for Carotenoids: Comparative Studies of Loading Ability, Storage Stability and in Vitro Release. Food Funct..

[41] Shafaa M.W.I., Diehl H.A., Socaciu C. (2007). The Solubilisation Pattern of Lutein, Zeaxanthin, Canthaxanthin and β-Carotene Differ Characteristically in Liposomes, Liver Microsomes and Retinal Epithelial Cells. Biophys. Chem..

[42] Petyaev I.M., Zigangirova N.A., Tsibezov V.V., Morgunova E.Y., Bondareva N.E., Kyle N.H., Bashmakov Y.K. (2018). Association with Monoclonal Antibody Promotes Intracellular Delivery of Lycopene. Monoclon. Antib. Immunodiagn. Immunother..

[43] Borel P., Desmarchelier C. (2018). Bioavailability of Fat-Soluble Vitamins and Phytochemicals in Humans: Effects of Genetic Variation. Annu. Rev. Nutr..

[44] Li B., Vachali P., Chang F.Y., Gorusupudi A., Arunkumar R., Shi L., Rognon G.T., Frederick J.M., Bernstein P.S. (2022). HDL Is the Primary Transporter for Carotenoids from Liver to Retinal Pigment Epithelium in Transgenic ApoA-I−/−/Bco2−/− Mice. Arch. Biochem. Biophys..

[45] Bhosale P., Larson A.J., Frederick J.M., Southwick K., Thulin C.D., Bernstein P.S. (2004). Identification and Characterization of a Pi Isoform of Glutathione S-Transferase (GSTP1) as a Zeaxanthin-Binding Protein in the Macula of the Human Eye. J. Biol. Chem..

[46] Li B., Vachali P., Frederick J.M., Bernstein P.S. (2011). Identification of StARD3 as a Lutein-Binding Protein in the Macula of the Primate Retina. Biochemistry.

[47] Li B., Vachali P., Bernstein P.S. (2010). Human Ocular Carotenoid-Binding Proteins. Photochem. Photobiol. Sci..

[48] Bandara S., von Lintig J. (2022). Aster La Vista: Unraveling the Biochemical Basis of Carotenoid Homeostasis in the Human Retina. BioEssays.

[49] Maksimov E.G., Moldenhauer M., Shirshin E.A., Parshina E.A., Sluchanko N.N., Klementiev K.E., Tsoraev G.V., Tavraz N.N., Willoweit M., Schmitt F.J. (2016). A Comparative Study of Three Signaling Forms of the Orange Carotenoid Protein. Photosynth. Res..

[50] Hagemann M., Eisenhut M., Hackenberg C., Bauwe H. (2010). Pathway and Importance of Photorespiratory 2-Phosphoglycolate Metabolism in Cyanobacteria.

[51] Slonimskiy Y.B., Zupnik A.O., Varfolomeeva L.A., Boyko K.M., Maksimov E.G., Sluchanko N.N. (2022). A Primordial Orange Carotenoid Protein: Structure, Photoswitching Activity and Evolutionary Aspects. Int. J. Biol. Macromol..

[52] Slonimskiy Y.B., Egorkin N.A., Ashikhmin A.A., Friedrich T., Maksimov E.G., Sluchanko N.N. (2022). Reconstitution of the Functional Carotenoid-Binding Protein from Silkworm in E. Coli. Int. J. Biol. Macromol..

[53] Maksimov E.G., Zamaraev A.V., Parshina E.Y., Slonimskiy Y.B., Slastnikova T.A., Abdrakhmanov A.A., Babaev P.A., Efimova S.S., Ostroumova O.S., Stepanov A.V. (2020). Soluble Cyanobacterial Carotenoprotein as a Robust Antioxidant Nanocarrier and Delivery Module. Antioxidants.

[54] Sluchanko N.N., Slonimskiy Y.B., Egorkin N.A., Varfolomeeva L.A., Faletrov Y.V., Moysenovich A.M., Parshina E.Y., Friedrich T., Maksimov E.G., Boyko K.M. (2022). Silkworm Carotenoprotein as an Efficient Carotenoid Extractor, Solubilizer and Transporter. Int. J. Biol. Macromol..

[55] Feldman T.B., Yakovleva M.A., Arbukhanova P.M., Borzenok S.A., Kononikhin A.S., Popov I.A., Nikolaev E.N., Ostrovsky M.A. (2015). Changes in Spectral Properties and Composition of Lipofuscin Fluorophores from Human-Retinal-Pigment Epithelium with Age and Pathology. Anal. Bioanal. Chem..

[56] Moldenhauer M., Sluchanko N.N., Buhrke D., Zlenko D.V., Tavraz N.N., Schmitt F.J., Hildebrandt P., Maksimov E.G., Friedrich T. (2017). Assembly of Photoactive Orange Carotenoid Protein from Its Domains Unravels a Carotenoid Shuttle Mechanism. Photosynth. Res..

[57] Slonimskiy Y.B., Egorkin N.A., Friedrich T., Maksimov E.G., Sluchanko N.N. (2022). Microalgal Protein AstaP Is a Potent Carotenoid Solubilizer and Delivery Module with a Broad Carotenoid Binding Repertoire. FEBS J..

[58] Sluchanko N.N., Slonimskiy Y.B., Egorkin N.A., Varfolomeeva L.A., Kleymenov S.Y., Minyaev M.E., Faletrov Y.V., Moysenovich A.M., Parshina E.Y., Friedrich T. (2022). Structural Basis for the Carotenoid Binding and Transport Function of a START Domain. Structure.

[59] Sparrow J.R., Wu Y., Nagasaki T., Yoon K.D., Yamamoto K., Zhou J. (2010). Fundus Autofluorescence and the Bisretinoids of Retina. Photochem. Photobiol. Sci..

[60] Feng J., Chen X., Sun X., Wang F., Sun X. (2014). Expression of Endoplasmic Reticulum Stress Markers GRP78 and CHOP Induced by Oxidative Stress in Blue Light-Mediated Damage of A2E-Containing Retinal Pigment Epithelium Cells. Ophthalmic Res..

[61] Nordgaard C.L., Karunadharma P.P., Feng X., Olsen T.W., Ferrington D.A. (2008). Mitochondrial Proteomics of the Retinal Pigment Epithelium at Progressive Stages of Age-Related Macular Degeneration. Investig. Ophthalmol. Vis. Sci..

[62] Feldman T., Ostrovskiy D., Yakovleva M., Dontsov A., Borzenok S., Ostrovsky M. (2022). Lipofuscin-Mediated Photic Stress Induces a Dark Toxic Effect on ARPE-19 Cells. Int. J. Mol. Sci..

[63] Hütter E., Skovbro M., Lener B., Prats C., Rabøl R., Dela F., Jansen-Dürr P. (2007). Oxidative Stress and Mitochondrial Impairment Can Be Separated from Lipofuscin Accumulation in Aged Human Skeletal Muscle. Aging Cell.

[64] Ben-Shabat S., Itagaki Y., Jockusch S., Sparrow J.R., Turro N.J., Nakanishi K. (2002). Formation of a Nonaoxirane from A2E, a Lipofuscin Fluorophore Related to Macular Degeneration, and Evidence of Singlet Oxygen Involvement. Angew. Chem. Int. Ed..

[65] Shcheslavskiy V.I., Shirmanova M.V., Dudenkova V.V., Lukyanov K.A., Gavrina A.I., Shumilova A.V., Zagaynova E., Becker W. (2018). Fluorescence Time-Resolved Macroimaging. Opt. Lett..

[66] Pospíšil P., Prasad A., Rác M. (2019). Mechanism of the Formation of Electronically Excited Species by Oxidative Metabolic Processes: Role of Reactive Oxygen Species. Biomolecules.

[67] Semenov A.N., Yakimov B.P., Rubekina A.A., Gorin D.A., Drachev V.P., Zarubin M.P., Velikanov A.N., Lademann J., Fadeev V.V., Priezzhev A.V. (2020). The Oxidation-Induced Autofluorescence Hypothesis: Red Edge Excitation and Implications for Metabolic Imaging. Molecules.

[68] Hashimoto H., Uragami C., Yukihira N., Gardiner A.T., Cogdell R.J. (2018). Understanding/Unravelling Carotenoid Excited Singlet States. J. R. Soc. Interface.

[69] Murillo A.G., Hu S., Fernandez M.L. (2019). Zeaxanthin: Metabolism, Properties, and Antioxidant Protection of Eyes, Heart, Liver, and Skin. Antioxidants.

[70] Holz F.G., Fleckenstein M., Schmitz-Valckenberg S., Bird A.C., Holz F.G., Schmitz-Valckenberg S., Spaide R.F., Bird A.C. (2007). Evaluation of Fundus Autofluorescence Images. Atlas of Fundus Autofluorescence Imaging.

[71] Pole C., Ameri H. (2021). Fundus Autofluorescence and Clinical Applications. J. Ophthalmic. Vis. Res..

[72] Wu Y., Yanase E., Feng X., Siegel M.M., Sparrow J.R. (2010). Structural Characterization of Bisretinoid A2E Photocleavage Products and Implications for Age-Related Macular Degeneration. Proc. Natl. Acad. Sci. USA.

[73] Delori F.C., Goger D.G., Dorey C.K. (2001). Age-Related Accumulation and Spatial Distribution of Lipofuscin in RPE of Normal Subjects. Investig. Ophthalmol. Vis. Sci..

[74] Feldman T.B., Yakovleva M.A., Larichev A.V., Arbukhanova P.M., Radchenko A.S., Borzenok S.A., Kuzmin V.A., Ostrovsky M.A. (2018). Spectral Analysis of Fundus Autofluorescence Pattern as a Tool to Detect Early Stages of Degeneration in the Retina and Retinal Pigment Epithelium. Eye.

[75] Sauer L., Gensure R.H., Andersen K.M., Kreilkamp L., Hageman G.S., Hammer M., Bernstein P.S. (2018). Patterns of Fundus Autofluorescence Lifetimes in Eyes of Individuals with Nonexudative Age-Related Macular Degeneration. Investig. Ophthalmol. Vis. Sci..

[76] Dysli C., Schürch K., Pascal E., Wolf S., Zinkernagel M.S. (2018). Fundus Autofluorescence Lifetime Patterns in Retinitis Pigmentosa. Investig. Ophthalmol. Vis. Sci..

[77] Dysli C., Wolf S., Hatz K., Zinkernagel M.S. (2016). Fluorescence Lifetime Imaging in Stargardt Disease: Potential Marker for Disease Progression. Investig. Ophthalmol. Vis. Sci..

[78] Sauer L., Gensure R.H., Hammer M., Bernstein P.S. (2018). Fluorescence Lifetime Imaging Ophthalmoscopy: A Novel Way to Assess Macular Telangiectasia Type 2. Ophthalmol. Retin..

[79] Schweitzer D., Deutsch L., Klemm M., Jentsch S., Hammer M., Peters S., Haueisen J., Müller U.A., Dawczynski J. (2015). Fluorescence Lifetime Imaging Ophthalmoscopy in Type 2 Diabetic Patients Who Have No Signs of Diabetic Retinopathy. J. Biomed. Opt..

[80] Sadda S.V.R., Borrelli E., Fan W., Ebraheem A., Marion K.M., Harrington M., Kwon S. (2019). A Pilot Study of Fluorescence Lifetime Imaging Ophthalmoscopy in Preclinical Alzheimer’s Disease. Eye.

