# Supplementary material for: Protein-Mediated Carotenoid Delivery Suppresses the Photoinducible Oxidation of Lipofuscin in Retinal Pigment Epithelial Cells
Source: Antioxidants (Basel). 2023 Feb 8;12(2):413. doi: 10.3390/antiox12020413 (PMC9952040; doi:10.3390/antiox12020413)
Supplement: Supplementary file 1 [file antioxidants-12-00413-s001.zip › antioxidants-2182406-supplementary.pdf]

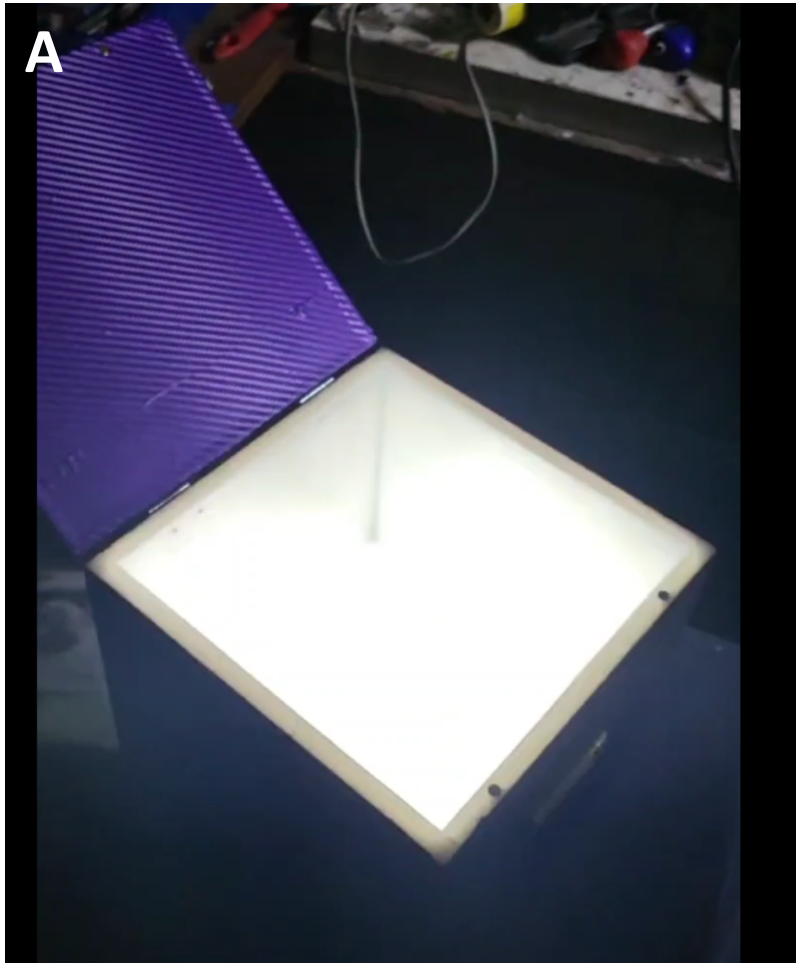

B

- Irradiation exposure time: 18 hours
- Light intensity 0.28-0.30 mW/cm^2

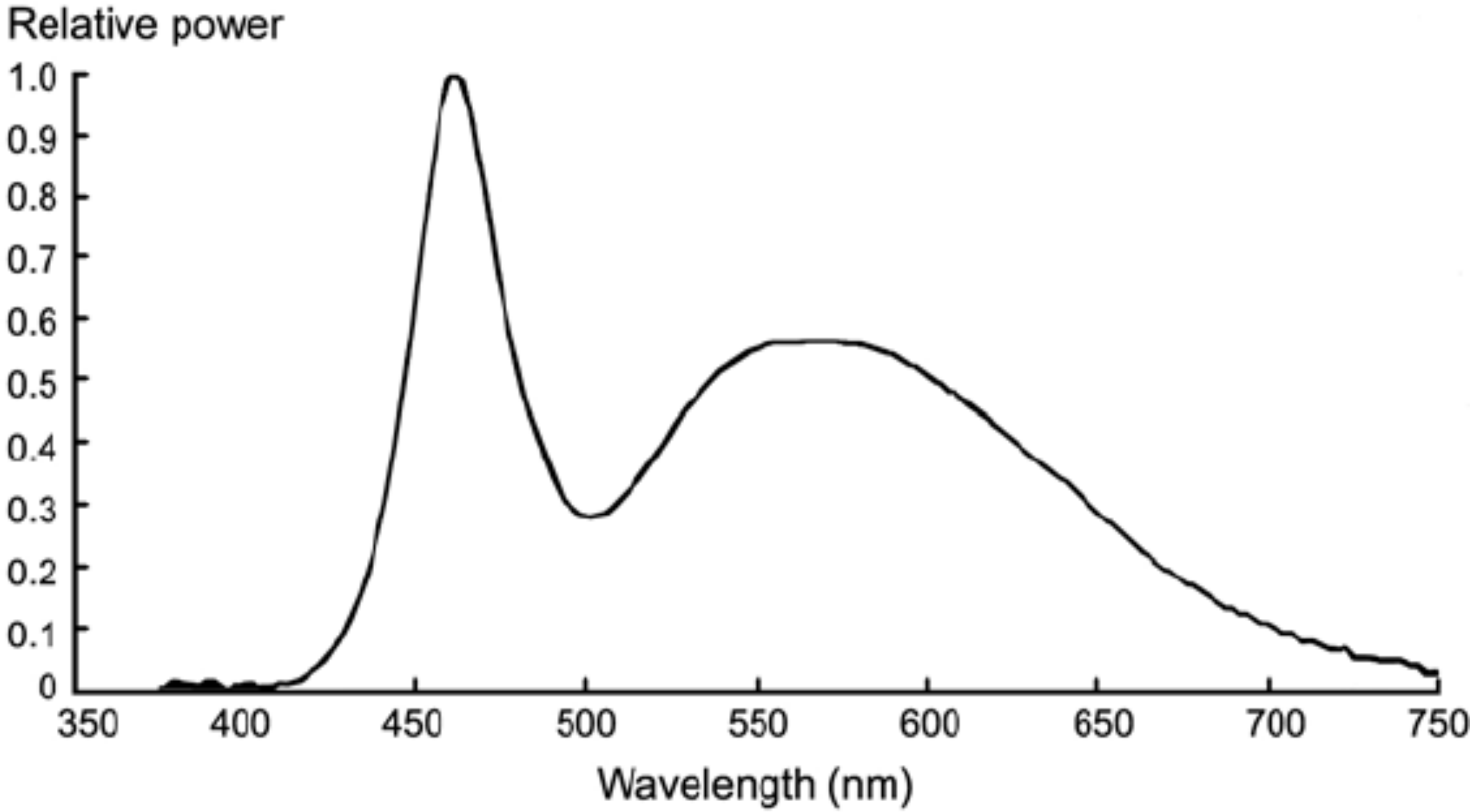

Supplementary Figure S1. A – photograph of the diode light lamp inside the plastic container, which was put into CO_2-incubator; B – spectrum of the diode lamp light, which was used in the experiments.

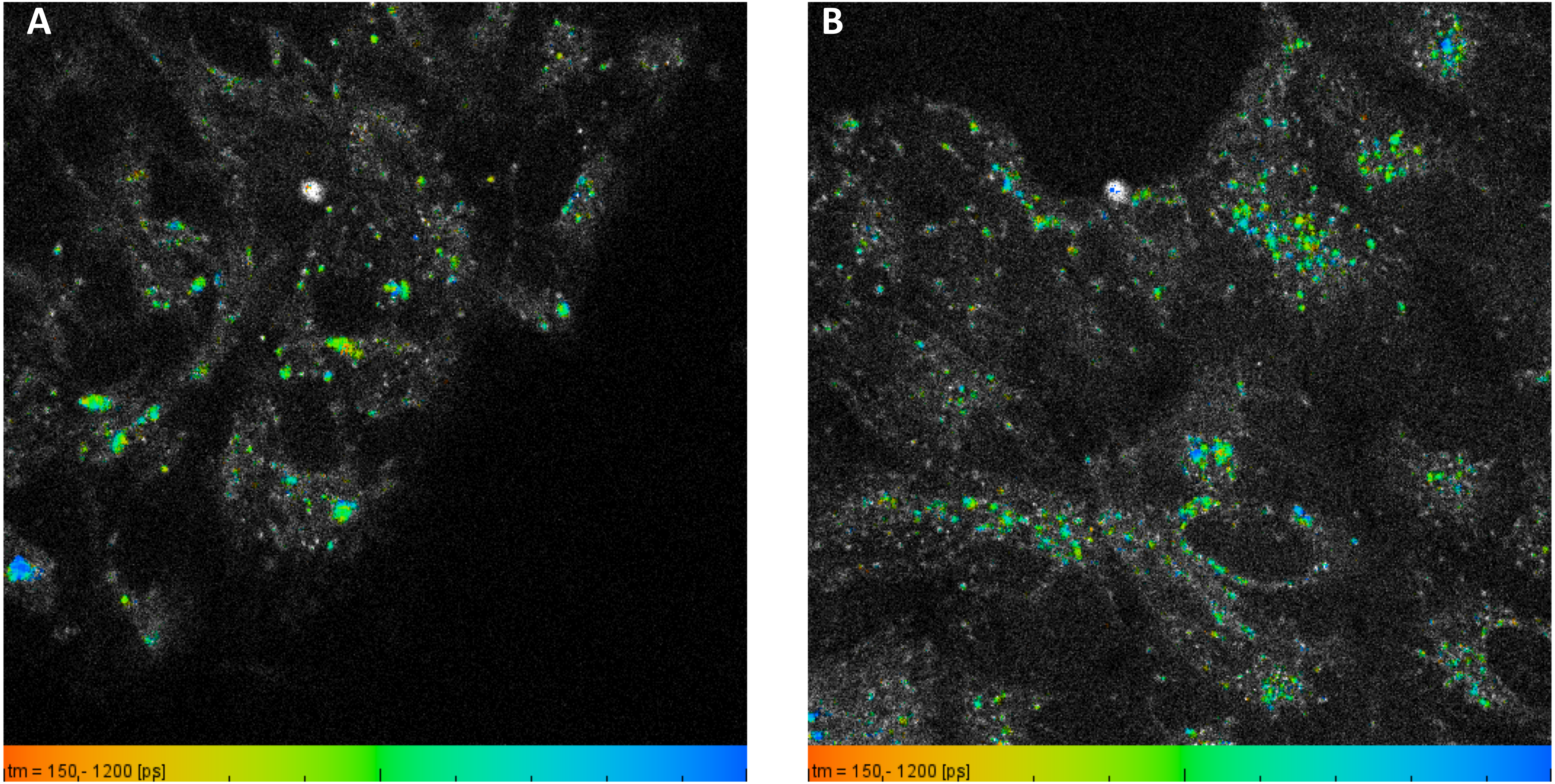

Supplementary Figure S2. Autofluorescence FLIM images of intact ARPE-19 cells: A – non-irradiated; B – irradiated with white diode lamp light. Brightness and contrast greatly (1000 times) increased.
